# Taurine-Dominated Feeding Attractant Mixture Induces Efficient Foraging in *Neptunea cumingii*

**DOI:** 10.3390/biology14111627

**Published:** 2025-11-19

**Authors:** Deliang Li, Wenjing Ren, Pengcheng Sun, Zhaoyu He, Fenghe An, Lei Gao, Xueshu Zhang, Ming Li

**Affiliations:** 1 Liaoning Key Laboratory of Marine Animal Immunology & Disease Control, Dalian Ocean University, Dalian 116023, China; deliangli@outlook.com (D.L.); rwj1012423@outlook.com (W.R.); pengchengsun123@outlook.com (P.S.); zhaoyuhe0130@outlook.com (Z.H.); gaolei@dlou.edu.cn (L.G.); 2 Dalian Key Laboratory of Aquatic Animal Disease Prevention and Control, Dalian Ocean University, Dalian 116023, China; 3 Freshwater Fisheries Research Center, Chinese Academy of Fishery Sciences, Wuxi 214081, China; anfenghe@ffrc.cn; 4 Zoneco Group Co., Ltd., Dalian 116001, China; 5 College of Fisheries, Ocean University of China, Qingdao 266003, China

**Keywords:** *Neptunea cumingii*, attractant, taurine, foraging behavior

## Abstract

The *Neptunea cumingii* (*N. cumingii*) fishing industry has long relied on expensive and perishable skate (*Raja porosa*) meat as bait. The unknown chemical attraction mechanism has hindered the development of artificial alternatives. This study employed untargeted metabolomics to analyze the chemical composition of skate meat and combined quantitative behavioral analysis to identify four key attractant compounds. These compounds were taurine, glutamate (Glu), inositol, and lactate. A standardized behavioral assessment system was established using the three parameters of response time, displacement distance, and movement velocity. This system enabled precise quantification of attraction efficacy. Concentration-gradient experiments determined the optimal concentration for all four compounds as 0.1 M. Taurine exhibited the strongest single-compound activity. It reduced response time by 50% and increased displacement distance by 164.5%. The mixture of four compounds at 0.1 M produced significant synergistic effects. The mixture achieved a comprehensive score of 93.6. This score approached that of natural skate meat at 94.8. All behavioral parameters improved by over 69% compared to the best single compound. These findings reveal the key attractant components in skate meat. They provide a scientific basis for developing efficient and stable artificial attractants. This research holds substantial value for promoting sustainable development of the *N. cumingii* fishing industry.

## Simple Summary

The fishing industry for *Neptunea cumingii* relies on expensive and perishable skate meat as bait. The chemical basis of this attraction was unknown, preventing development of cost-effective alternatives. This study aimed to identify the specific chemicals in skate meat that attract the snails. We analyzed skate meat composition and tested snail behavioral responses to different compounds. Four key attractant chemicals were identified, including taurine, glutamate, inositol, and lactate. When these four chemicals were combined at optimal concentrations, the mixture reached ninety percent of the effectiveness of natural skate meat and performed sixty-nine percent better than any single compound alone. These findings identify the key chemical components responsible for attraction and show that synthetic formulations can serve as effective alternatives to natural bait. This research provides the foundation for producing affordable and stable artificial attractants, which could reduce fishing costs and support economically sustainable fishing practices.

## 1. Introduction

*Neptunea cumingii* (*N. cumingii*) is an economically important shellfish species in the North Pacific region [1,2,3]. Its complex reproductive biology and low larval survival rates have prevented breakthroughs in artificial cultivation technology [4,5,6,7]. The market supply relies entirely on wild resource harvesting [2]. Among the various fishing methods, trap-based techniques utilizing foraging behavior have become the primary commercial approach [8]. This method offers simple operation, high efficiency, and minimal ecological impact [9]. Current trapping operations commonly use fresh skate (*Raja porosa*) meat as bait. This practice faces multiple challenges including high procurement costs, rapid spoilage, difficult storage and transportation, and unstable supply [10]. These limitations increase production costs, reduce economic benefits, and threaten sustainable fishery development [9,11,12]. Developing artificial attractants with stable performance, low cost, and reliable sources has become a critical technical requirement for the industry [13,14,15,16]. The key to solving this technical challenge lies in identifying and understanding the chemical attraction mechanisms of *N. cumingii*.

Chemical signals mediate foraging behavior as a key survival strategy in aquatic animals [17]. Predators detect chemical molecules released by prey through sensory receptors. Neural signal transduction then triggers directional movement and feeding responses. Identifying these key chemical molecules is central to attractant development. Recent advances in metabolomics and related analytical technologies have significantly progressed chemical attractant research in aquatic animals [18,19]. Research on fish has identified amino acids, nucleotides, and betaine as effective attractants [20,21]. These compounds demonstrate significant effects in promoting fish feeding and improving feed conversion efficiency [22,23]. Gastropod mollusks possess well-developed chemoreceptor systems with tentacles and osphradium as primary sensory organs [13]. Attractant research on gastropods lags far behind fish studies [24]. A few studies have reported that glutamate (Glu) stimulates foraging behavior in *Rapana venosa* larvae. The response mechanisms of *N. cumingii* to potential attractants remain poorly understood. Investigation of attraction-active substances and chemoreception mechanisms in *N. cumingii* is essential for developing cost-effective artificial attractants and promoting sustainable fishery development.

This study employed untargeted metabolomics to analyze the chemical composition of skate meat and identify key attractant components. A multidimensional screening system and standardized behavioral assessment method were established to systematically examine individual and synergistic effects of candidate compounds. Computer vision technology enabled precise quantification of foraging behavior and provided an objective basis for evaluating attraction activity. The findings provide a scientific foundation for developing artificial attractants for *N. cumingii* and hold significant value for improving fishing efficiency and promoting sustainable industry development.

## 2. Materials and Methods

### 2.1. Experimental Animals

*N. cumingii* specimens were collected from Changhai County waters in Dalian, Liaoning Province, China. Healthy individuals with shell lengths of 10.0 ± 1.0 cm (measured as the straight-line distance from the apex to the farthest point of the aperture) were selected for experiments. Specimens were maintained in plastic tanks (1.0 m × 0.5 m × 0.3 m) with filtered seawater for at least 7 days before the experiments. Water temperature was maintained at 12.0 ± 2.0 °C. Salinity was kept at 30.0 ± 1.0 ‰ and pH at 8.1 ± 0.1. Continuous aeration ensured sufficient dissolved oxygen. Seawater was completely replaced every 48 h to maintain water quality. Stocking density was limited to a maximum of 15 individuals per tank. No feeding was provided during the acclimation period. All experimental procedures followed the guidelines of the Animal Ethics Committee of Dalian Ocean University.

### 2.2. Skate Meat Processing and Aqueous Extract Preparation

Skate meat was obtained from Zhangzidao Breeding Farm. After removing skin and connective tissue, the skate meat was cut into small pieces and immediately protected from light to prevent metabolite photodegradation. All procedures were performed under minimal light exposure to protect metabolites. To detect all metabolites present in skate tissue, skate meat samples (5.0 g) were directly processed without adding water. The samples were flash-frozen in liquid nitrogen and ground into fine powder using a tissue grinder (Tissuelyser-24, Jingxin, Shanghai, China). Three biological replicates were prepared for each group and stored at −80 °C for subsequent metabolomic analysis to capture the complete metabolite profile of skate tissue.

**Figure 1:**
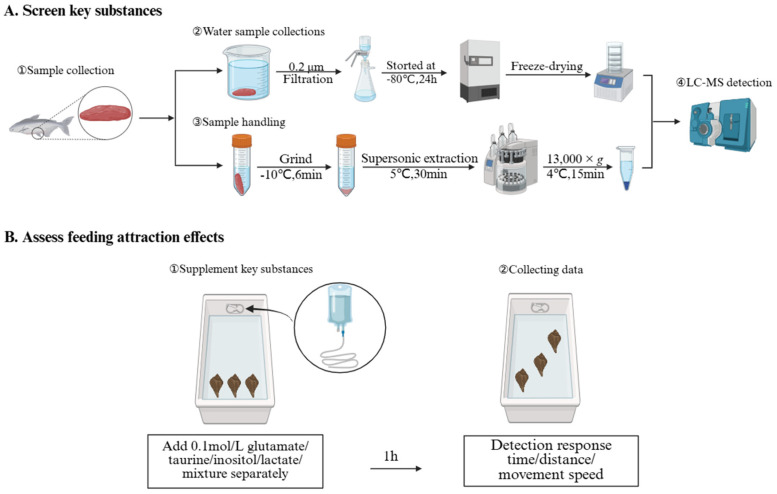
Flowchart of key substance screening and trapping data collection. (**A**) Water sample collection, sample processing, and LC-MS detection workflow. (**B**) Key substance supplementation experiment and feeding behavior data collection workflow.

To prepare aqueous extracts containing water-soluble metabolites, skate meat samples (5.0 g wet weight) were placed in 500 mL deionized water in sealed containers. The samples were soaked at 4 °C in darkness for 12 h to allow for sufficient release of water-soluble metabolites while minimizing degradation, with three biological replicates per treatment. After soaking, the supernatant was carefully collected and filtered through 0.22 μm membrane filters to remove tissue debris and microorganisms. The filtrate was then distributed into 100 mL centrifuge tubes (approximately 80 mL per tube) and pre-frozen at −80 °C for 2 h to ensure complete solidification prior to lyophilization. Subsequently, samples were freeze-dried continuously for 72 h using a freeze dryer (Christ Alpha 1-2 LD plus, Christ, Osterode am Harz, Germany) under a condenser temperature of −50 °C and <10 Pa vacuum pressure until all water was removed, yielding a yellow lyophilized powder (Figure 1A). The lyophilized powder was immediately sealed and stored at −80 °C for subsequent metabolomic analysis. These aqueous extracts containing bioavailable water-soluble metabolites released from skate tissue were subjected to metabolomic analysis to identify the key compounds capable of triggering chemoreceptor responses in *N. cumingii*.

### 2.3. Untargeted Metabolomic Analysis

Two sample groups were prepared for metabolomic comparison. The tissue samples (T group) represented direct muscle tissue composition, and the aqueous extract samples (W group) represented water-soluble metabolites released during the 12-h soaking. Aqueous extracts and skate meat samples (*n* = 3) were sent to Wuhan Benagen Technology Co., Ltd. (Wuhan, China), for untargeted metabolomic analysis. Samples were analyzed using both gas chromatography-mass spectrometry (GC-MS) and ultra-high-performance liquid chromatography coupled with quadrupole time-of-flight mass spectrometry (UHPLC-Q-TOF MS) platforms. GC-MS detected volatile and semi-volatile metabolites while LC-MS analyzed non-volatile compounds, providing complementary and comprehensive metabolite coverage. Data were processed through peak alignment, retention time correction, and peak area extraction. Metabolites were identified using the BiotreeDB (V3.0) database. Principal component analysis (PCA) and orthogonal partial least squares discriminant analysis (OPLS-DA) were performed to evaluate differences between groups. This comparison identified candidate compounds with high tissue abundance and efficient water release, which were subsequently validated for attractant activity through foraging behavioral assays.

### 2.4. Foraging Behavior Assay

A behavioral observation tank (1.0 m × 0.3 m × 0.3 m) was filled with natural seawater to a depth of 20 cm. Water temperature was maintained at 12.0 ± 2.0 °C and salinity at 30.0 ± 1.0 ‰. A water pump maintained a flow velocity of 0.5 cm/s to simulate natural conditions [25]. Snails were fasted for 96 h before the experiments to ensure a consistent foraging state. During the experiments, the snails were placed at one end of the tank. Test solutions included skate aqueous extract (Experiment) and four candidate compounds (taurine, Glu, inositol, and lactate). Each compound was tested at four concentration gradients (10^−3^ M, 10^−2^ M, 10^−1^ M, and 1.0 M). A mixture (Mix) containing all four compounds at 0.1 mol·L^−1^ was also tested. All test solutions were released at 1.0 mL/min through a micropump system at a position 70 cm upstream from the starting point. The blank control group received equal volumes of artificial seawater (*n* = 5). Each treatment was replicated three times using independent tanks, with five individuals per tank. Each experiment lasted 60 min. All experiments were conducted under consistent ambient lighting conditions. The camera (Go Pro, San Mateo, CA, USA) was mounted on a fixed bracket directly above the tank center to ensure complete coverage of the experimental area. Continuous video recording at 5-s intervals was used to track the movement trajectories of *N. cumingii* throughout the 60-min experimental period (Figure 1B).

### 2.5. Collection and Analysis of Behavioral Data

This study established a three-parameter behavioral assessment system centered on response time, displacement distance, and movement speed to quantitatively measure the behavioral responses of *N. cumingii* to different attractants. Response time was operationally defined as the time interval from attractant release to when the snail moved a minimum straight-line distance of 2 cm toward the signal source(s). Displacement distance was recorded by tracking individual movement trajectories from the starting position to the experimental endpoint (pixels). Movement speed was calculated by dividing total displacement distance by the corresponding time duration (pixels/s). An automated tracking system based on computer vision was developed to accurately quantify snail movement behavior. The system employed the YOLOv11 deep learning model for object detection and integrated the DeepSORT multi-object tracking algorithm to establish individual movement trajectories (Supplementary Materials Code S1). The system processed experimental videos frame-by-frame and automatically extracted key parameters including timestamps, tracking IDs, and center coordinates. Data were exported in CSV format. Behavioral outcomes were categorized according to final individual positions as successful type (arrival and residence near the chemical signal source with distance < 10 cm) and unsuccessful type (failure to reach the signal source or exhibition of avoidance behavior). These quantitative parameters were integrated to construct a comprehensive behavioral assessment framework for comparative analysis of *N. cumingii* responses to different attractants under standardized experimental conditions.

### 2.6. Construction Standards for Attractant Efficacy Scoring System

This scoring system established quantitative evaluation criteria based on snail feeding behavioral parameters. The improvement rate or enhancement rate was calculated for relative changes in each indicator (Table 1). Graded scoring criteria were set according to data distribution patterns. Comprehensive scores were calculated through the weighted average method (response time: 0.4; movement distance: 0.35; movement speed: 0.25). Four efficacy levels were designated to provide objective evidence for attractant screening. Weight coefficients were determined based on measurement reliability and biological significance established in foraging behavior models [26,27], with thresholds calibrated against natural bait responses following standard protocols [28].

Comprehensive score = Response time score × 40% + Movement distance score × 35% + Movement speed score × 25%.

### 2.7. Statistical Analysis

All data are expressed as means ± standard deviations (means ± SDs). Statistical analyses were performed using SPSS 22.0. Graphs were generated using OriginPro 8.0. Multiple-group comparisons were conducted using one-way analysis of variance (ANOVA). Pairwise comparisons between groups employed Tukey’s HSD test. Differences were considered significant if *p* < 0.05 and highly significant if *p* < 0.01. All experiments were replicated three times.

## 3. Results

### 3.1. Effects of Skate Meat on Foraging Behavior of N. cumingii

**Figure 2:**
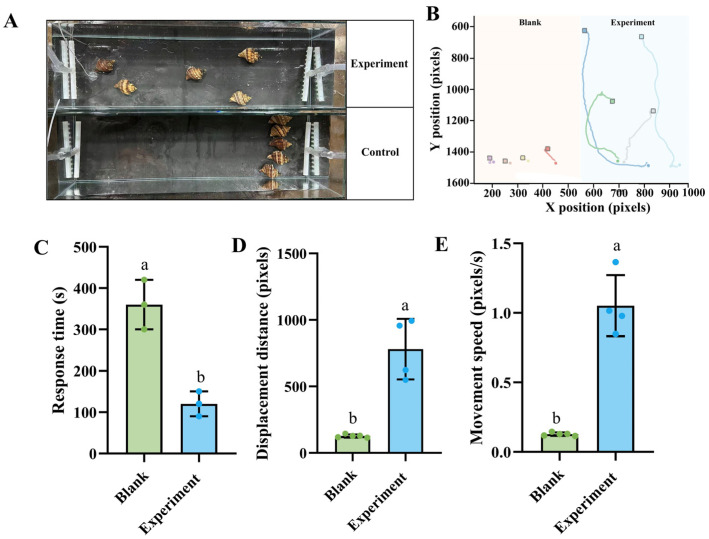
Attractant effect of skate meat on *N. cumingii*. (**A**) Final position distribution of *N. cumingii*. Observations were made 0.5 h after skate meat attractant treatment (Experiment) and control treatment without attractant (Blank). (**B**) Crawling path diagrams of *N. cumingii*. Movement trajectories were analyzed within 1 h after skate meat attractant treatment (Experiment, blue background) and control treatment (Blank, pink background). Different colored trajectories and squares represent different individual. (**C**–**E**) Behavioral parameters of *N. cumingii*. Response time (**C**), displacement distance (**D**), and maximum crawling speed (**E**) were measured under skate meat attractant treatment (Experiment) and control treatment (Blank). Different lowercase letters indicate significant differences between groups (*p* < 0.05).

Skate meat treatment significantly altered the spatial distribution and movement patterns of *N. cumingii*. Individuals in the skate meat treatment group exhibited obvious directional chemotactic behavior with movement trajectories converging toward the signal source and displaying aggregated distribution patterns (Figure 2A). Representative movement trajectories illustrated distinct behavioral differences between the two groups. Organisms in the treatment group showed linear, directed paths oriented toward the chemical source, while control individuals displayed irregular, multidirectional wandering patterns with no apparent orientation (Figure 2B). Further behavioral analysis revealed that response time in the skate meat treatment group decreased significantly to 120 s, representing a 70% reduction compared to the control group. Displacement distance reached 800 pixels, showing a 6.2-fold increase. Movement speed reached 1.1 pixels/s, demonstrating an 8.5-fold enhancement compared to the control group (Figure 2C–E). These results indicate that chemical signals from skate meat significantly enhance foraging behavioral responses in *N. cumingii*.

### 3.2. Screening of Key Metabolites Through Metabolomics

**Figure 3:**
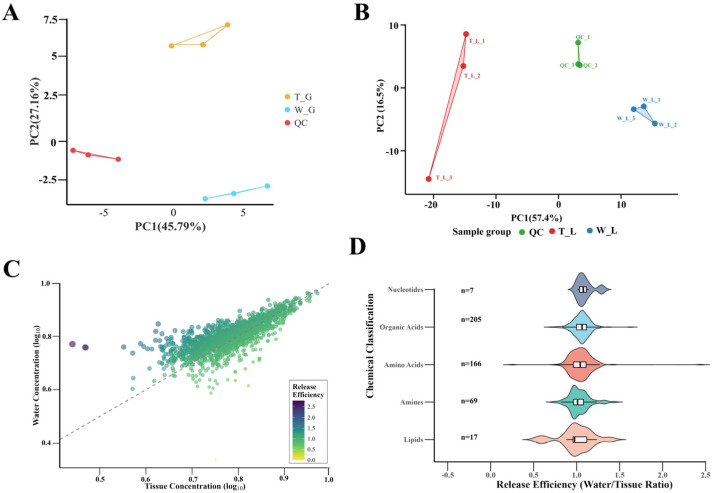
Analysis of metabolite release characteristics from skate meat. (**A**) Principal component analysis of GC-MS metabolomic data showing separation of quality control samples (QC), tissue samples (T_G), and water samples (W_G). (**B**) Principal component analysis of LC-MS metabolomic data showing separation of quality control samples (QC), tissue samples (T_L), and water samples (W_L). (**C**) Scatter plot of metabolite release efficiency with tissue concentration on x-axis and water concentration on y-axis, with color indicating release efficiency (water/tissue concentration ratio). (**D**) Release efficiency distribution of metabolites across different chemical categories including carbohydrates, nucleotides, organic acids, amines, amino acids, and lipids.

Metabolomic analysis was performed on skate meat extract to identify the key compounds. Principal Component Analysis (PCA) showed clear separation between the experimental group (T) and control group (W) in both GC-MS and LC-MS data, indicating good discrimination and reliability of the metabolomic data (Figure 3A,B). Release efficiency analysis revealed significant differences in the release capacity of different metabolite categories. Amino acid compounds exhibited the highest release efficiency. Taurine and Glu showed significantly higher release efficiency than other amino acids (Figure 3C,D). GC-MS quantitative analysis demonstrated that inositol and lactate had the highest concentrations among the detected water-soluble metabolites (Table 2). Based on the two key parameters of release efficiency and aqueous content, four candidate attractant compounds were finally selected. These compounds included taurine, Glu, inositol, and lactate. This selection provided the foundation for subsequent activity validation.

### 3.3. Effects of Individual Key Metabolites on Foraging Behavior of N. cumingii

**Figure 4:**
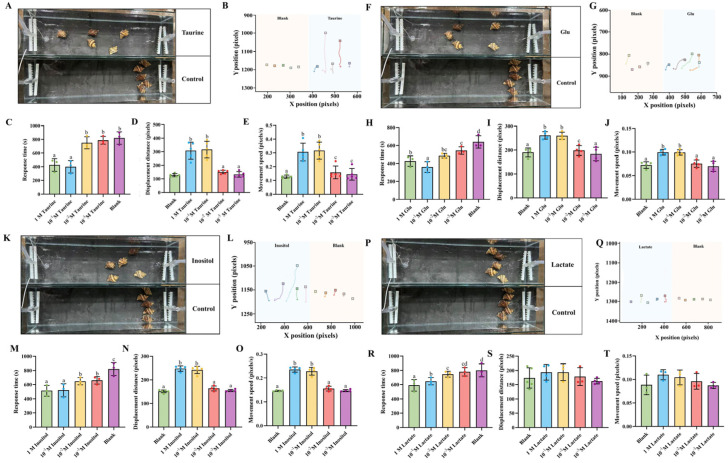
Attractant efficacy of four compound concentration gradients for *N. cumingii*. Behavioral response analysis of *N. cumingii* after 1-h treatment with four candidate attractant compounds (taurine, Glu, inositol, lactate) at different concentrations (10^−3^ M, 10^−2^ M, 10^−1^ M, 1.0 M). (**A**,**F**,**K**,**P**) Experimental photographs of treatment groups at optimal concentration (0.1 M) and blank control groups for each compound. (**B**,**G**,**L**,**Q**) Movement trajectory diagrams of *N. cumingii* with green trajectories representing treatment groups and gray trajectories representing blank control groups. Different colored trajectories and squares represent different individual. (**C**–**E**) Behavioral parameters under taurine concentration gradient: response time (**C**), displacement distance (**D**), and movement speed (**E**). (**H**–**J**) Behavioral parameters under Glu concentration gradient: response time (**H**), displacement distance (**I**), and movement speed (**J**). (**M**–**O**) Behavioral parameters under inositol concentration gradient: response time (**M**), displacement distance (**N**), and movement speed (**O**). (**R**–**T**) Behavioral parameters under lactate concentration gradient: response time (**R**), displacement distance (**S**), and movement speed (**T**). Bar charts show comparison results between different concentration treatment groups and control groups. Different lowercase letters indicate significant differences between groups (*p* < 0.05).

To validate the attractant activity of the four candidate compounds identified through metabolomics screening, a concentration gradient method (10^−3^ M to 1.0 M) was employed to evaluate their dose–response relationships. All four compounds exhibited optimal attractant effects at a concentration of 0.1 M. Activity intensity varied significantly among compounds. Taurine demonstrated the strongest attractant activity. At 10^−3^ M and 10^−2^ M concentrations, there were no significant differences in behavioral parameters compared to the control group. The 0.1 M treatment group showed a response time of 50% of the control group’s response time. Both movement speed and displacement distance increased by 2.64-fold compared to the control group. The parameter values decreased somewhat at a concentration of 1.0 M compared to the 0.1 M group (Figure 4A–E). Glu showed no significant attractant effect at low concentrations (10^−3^ M and 10^−2^ M). The 0.1 M treatment group showed a 44% reduction in response time and a 1.33-fold increase in displacement distance compared to the control group. The behavioral parameters at a concentration of 1.0 M showed no significant differences from those of the 0.1 M group (Figure 4F–J). Inositol attractant activity increased in a concentration-dependent manner. The 0.1 M treatment group showed a response time of 520 s. The behavioral parameters were 1.87-fold higher than those of the control group. The parameters at a concentration of 1.0 M showed no significant differences from those of the 0.1 M group (Figure 4K–O). Lactic acid exhibited low attractant activity across the entire concentration range. Response time only decreased to 580 s even at the optimal concentration of 0.1 M. Parameter improvements were minimal at 1.2-fold (Figure 4P–T).

### 3.4. Effects of Mixed Key Metabolites on Foraging Behavior of N. cumingii

**Figure 5:**
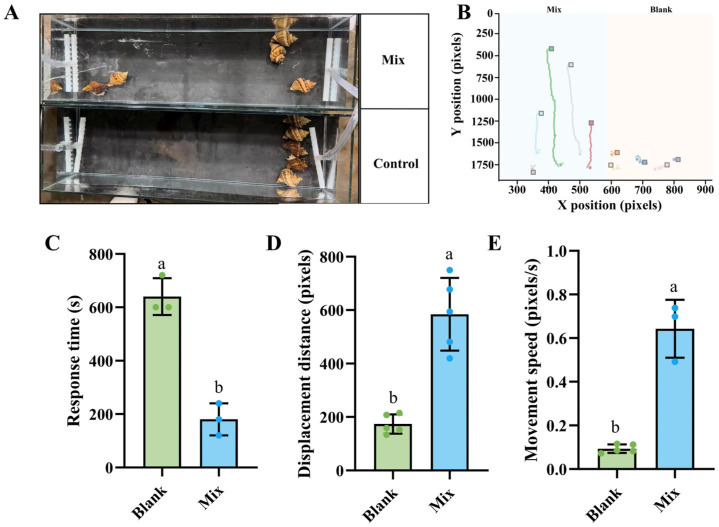
Attractant effect of four-substance mixture on *N. cumingii*. (**A**) Attractant effect of mixture on *N. cumingii*. Observations were made 1 h after mixture attractant treatment (Mix) and control treatment without attractant (Blank). (**B**) Crawling path diagrams of *N. cumingii*. Movement trajectories were analyzed within 1 h after mixture attractant treatment (Mix, blue background) and control treatment (Blank, pink background). Different colored trajectories and squares represent different individual. (**C**–**E**) Behavioral parameters of *N. cumingii*. Response time (**C**), displacement distance (**D**), and maximum crawling speed (**E**) were measured under mixture attractant treatment (Mix) and control treatment (Blank). Different lowercase letters indicate significant differences between groups (*p* < 0.05).

The synergistic effects of the four compounds in combination were further investigated based on individual compound performance. Individuals in the mixture group displayed intense directional aggregation behavior with movement trajectories converging toward the signal source. The control group maintained random dispersed distribution (Figure 5A,B). Behavioral analysis showed that the response time in the mixture group decreased to 190 s, representing a 69% reduction compared to the control group. Displacement distance reached 584 pixels, showing a 6.8-fold increase. Movement speed reached 0.64 pixels/s, demonstrating a 6.8-fold enhancement (Figure 5C–E). The effect of the mixed attractant clearly exceeded that of individual compound treatments, confirming the synergistic enhancement effect among multiple components.

### 3.5. Analysis of Attractant Efficacy Using Comprehensive Evaluation System

The established comprehensive evaluation system was employed to quantitatively assess all tested samples and provide a systematic comparison of attractant performance. The results showed that skate meat as a natural attractant performed best with a score of 94.8 points, demonstrating excellence across all three dimensions of response time, movement distance, and movement speed. Among individual chemical attractants, taurine exhibited the highest attractant effect at 55.4 points, followed by Glu at 38.5 points and inositol at 37.7 points. Lactate showed the weakest attractant effect at 21.7 points. The mixed attractant achieved excellent performance approaching that of the natural product through multi-component synergistic formulation with a score of 93.6 points (Figure 5 and Table 3). This evaluation system successfully quantified the differences in attractant effects among different attractants and validated the effectiveness of the multi-component synergistic formulation.

## 4. Discussion

This study identified four key attractant components from skate meat through a combined strategy of non-targeted metabolomics screening and behavioral validation [18,19]. These components included taurine, Glu, inositol, and lactate. Behavioral assessment revealed that the optimal concentration for all four compounds was 0.1 M. Taurine exhibited the strongest individual activity with a comprehensive score of 55.4 points. The four-component mixture produced significant synergistic effects with a score of 93.6 points, approaching the performance of natural skate meat at 94.8 points. This work identifies taurine-dominated bioactive formulations as a natural attractant for sustainable fisheries.

As a specialized amino acid containing a sulfonic acid group, taurine demonstrates a clear causal relationship between its molecular properties and behavioral responses in *N. cumingii* [29]. This study observed that taurine reduced response time by 50% from 400 s to 200 s. This rapid response originates from the unique mechanism through which taurine activates chemoreceptors. Taurine activates extrasynaptic α4β2δ GABA receptors at a low EC50 value of 57 μM [30]. The resulting depolarization current directly triggers neuronal impulses in the tentacles and osphradium of *N. cumingii*. The same receptors require millimolar concentrations of other ligands to produce similar effects [30]. Neural signals transmitted to the motor center after receptor activation explain the 164.5% increase in displacement distance. Taurine functions not only as a neurotransmitter but also as a muscle function regulator. It enhances contractile forces by increasing calcium ion release from the sarcoplasmic reticulum. Research on marine gastropods confirms that taurine can increase muscle contraction frequency by 35%, directly leading to enhanced crawling speed [31]. The 163% improvement in movement speed recorded in this study aligns with this mechanism. The material basis supporting these behavioral changes is the high release efficiency of taurine at 78.3%. Complete ionization of the sulfonic acid group at seawater pH ensures stability of the chemical gradient [32]. A diffusion coefficient of 7.5 × 10^−6^ cm^2^/s guarantees effective signal propagation distance [32]. Concentration effects further validate the receptor mechanism. The optimal effect at 0.1 M results from simultaneous activation of high and low affinity receptors, producing synergy. The decreased effect at 1.0 M occurs due to receptor saturation and desensitization [33,34]. This evidence demonstrates that taurine achieves efficient attraction through a triple mechanism of rapid chemoreceptor activation, enhanced muscle contraction, and maintenance of stable signals.

Glu, inositol, and lactate can all trigger initial responses in *N. cumingii*. Their effects fall far short of taurine. This difference reveals the intrinsic relationship between chemical attraction efficacy and metabolic function [26]. The concentration-dependent responses (Figure 5) reflect distinct molecular mechanisms, as receptor-mediated compounds (Glu and inositol) require optimal ligand concentrations for effective signaling but desensitize at high doses, whereas lactate, lacking specific receptors, shows minimal concentration sensitivity [35,36,37]. The ability of Glu to reduce response time by 44% indicates activation of the sensory process. The displacement distance increase of only 33% suggests a lack of capacity to maintain directional movement. This difference stems from complex regulation of the Glu receptor system. NMDA receptors require co-activation by Glu and glycine and exhibit voltage-dependent Mg^2+^ blockade [35,38]. This makes the receptor activation threshold far higher than that of the taurine GABA system [39]. Protein metabolism in adult *N. cumingii* has stabilized. The demand for Glu as a nitrogen metabolism precursor has decreased. During their rapid growth periods, juveniles show greater sensitivity to such nutritional signals [40]. Inositol demonstrates concentration-dependent enhancement with parameters improving 1.87-fold at 0.1 M. Its effect remains markedly weaker than taurine. Inositol regulates energy metabolism through the phosphatidylinositol pathway [36,41]. PLC hydrolysis of PIP2 produces IP3, triggering calcium ion release that is crucial in crustaceans [25]. *N. cumingii*, as a slow-moving predator, has a metabolic rate far lower than active crustaceans. Its dependence on rapid energy mobilization systems is reduced. Lactate shows the weakest effect with only 1.2-fold improvement. Fish can produce rapid responses to lactate through gill chemoreceptors [37]. The feeding mode of *N. cumingii* using radula scraping does not require rapid assessment of prey physiological status. The common characteristic of these three compounds is that they function as intracellular metabolic intermediates [42]. They are passively released upon tissue damage rather than actively secreted. They lack the specificity and stability required for chemical signaling. The limited response of *N. cumingii* to these metabolites reflects its feeding strategy as a benthic carnivore. It preferentially recognizes stable and reliable protein markers rather than transient metabolic signals.

The synergistic enhancement after mixing the four compounds with a comprehensive score of 93.6 points approaching natural skate meat at 94.8 points reveals that the complexity of marine chemical communication lies not in the intensity of a single signal but in functional complementarity and spatiotemporal integration of multiple components [43]. The mixture reduced response time by 69% and increased displacement distance by 579%. This significant improvement exceeds simple addition of individual components [44]. The shortened response time with enhanced displacement and speed reflects two-stage chemotaxis. Rapid signal detection triggers directional movement, while sustained locomotion requires continuous metabolic and muscular support. Enhanced chemosensory detection through complementary diffusion dynamics and dual receptor activation accounts for the faster responses. Synergy between taurine and Glu first manifests in complementarity of diffusion dynamics. The diffusion coefficient of Glu at 9.1 × 10^−6^ cm^2^/s is slightly higher than that of taurine at 7.5 × 10^−6^ cm^2^/s. This forms a dual-layer concentration gradient. Rapid diffusion of Glu provides far-field signals guiding initial orientation. The stable gradient of taurine maintains sustained chemotaxis [32]. At the receptor level, the two activate different neural pathways producing signal amplification. Taurine activates GABA receptors, producing inhibitory modulation. Glu activates ionotropic receptors producing excitatory input. This excitatory-inhibitory balance prevents adaptive decline of a single system [45]. Although inositol and lactate show limited individual effects, they play important auxiliary roles in the mixed system. Inositol mobilizes intracellular calcium stores through the IP3 pathway, enhancing taurine-induced muscle contraction. Lactate functions as a metabolic state indicator. Its presence suggests fresh prey tissue, increasing the credibility of chemical signals [42]. Research demonstrates that feeding behavior in marine animals relies on “across-fiber pattern” coding. Combined signals from simultaneous activation of multiple chemoreceptors produce responses 2- to 10-fold higher than single components [44]. This nonlinear enhancement also involves metabolic interactions. Glu can convert to GABA enhancing taurine receptor activation. Energy metabolism regulated by inositol supports sustained motor output. The presence of lactate may lower sensory thresholds. Multi-component synergy through precise coordination across receptor, metabolic, and spatiotemporal dimensions reconstructs the composite chemical information characteristics of natural bait, providing a mechanistic foundation for developing efficient artificial attractants.

Economic analysis indicates that the optimized bait mixture demonstrates significant cost advantages in commercial aquaculture operations. Although the unit cost of the mixture is approximately twice that of skate meat, only 20% of the mixture mass is required to achieve equivalent attraction efficacy. This results in an approximately 59% reduction in bait costs. The ambient temperature stability of the mixture eliminates cold storage requirements and provides an extra 10% savings in total costs. For a commercial operation with 500-ton annual catch production, this strategy can reduce total skate meat bait costs from approximately $66,000 to $36,000 per year. This represents annual savings of approximately $30,000 and a 45% cost reduction. The method also eliminates the strong odor and spoilage issues associated with fresh skate meat and significantly improves working conditions. These encouraging preliminary results warrant further validation at larger scales to confirm the long-term economic sustainability and applicability of this bait strategy.

## 5. Conclusions

This study successfully identified four attractant-active compounds for *N. cumingii* through the integration of non-targeted metabolomics screening and quantitative behavioral evaluation. These compounds included taurine, Glu, inositol, and lactate. The optimal effective concentration of 0.1 M was established. Taurine exhibited the best performance in individual testing with a comprehensive score of 55.4 points. This superior performance was attributed to its high release efficiency at 78.3%, chemical stability, and multi-target neuromodulatory mechanisms. The mixture of four compounds produced significant synergistic enhancement with a score of 93.6 points, approaching the performance of natural skate meat at 94.8 points. This confirmed that multi-component chemical signals can reconstruct the composite chemical characteristics of natural bait through synergistic actions involving receptor complementarity, spatiotemporal gradient construction, and metabolic regulation. The standardized behavioral assessment system established in this study achieved precise quantification of attractant efficacy and provides scientific evidence for optimization of artificial attractant formulations for *N. cumingii*.

## Figures and Tables

**Table 1:** Attractant scoring criteria.

Category	Content	Standard Formula	Weight (%)
Improvement rate calculation	Response time improvement rate	(Control Value—Experimental Value)/Control Value × 100%	

	Movement distance enhancement rate	(Experimental Value—Control Value)/Control Value × 100%	

	Movement speed enhancement rate	(Experimental Value—Control Value)/Control Value × 100%	

Response time score	≥70%	100 points	Weight: 40
	50–69%	70–99 points (linear interpolation)	
	30–49%	40–69 points (linear interpolation)	
	<30%	<40 points	
Movement distance/Speed score	≥700%	100 points	Weight: 35/25
	200–699%	60–99 points (linear interpolation)	
	100–199%	30–59 points (linear interpolation)	
	<100	<30	
Effect level classification	Excellent	≥90 points	
	Good	70–89 points	
	Fair	50–69 points	
	Poor	<50 points	

**Table 2:** Metabolomic data analysis. Metabolomic analysis of skate meat soaking water samples was performed using gas chromatography-mass spectrometry (GC-MS) and liquid chromatography-mass spectrometry (LC-MS). GC-MS detected volatile and semi-volatile metabolites in positive ion mode (POS). LC-MS detected non-volatile metabolites in both positive ion mode (POS) and negative ion mode (NEG). The first three columns list the top 5 metabolites ranked by relative abundance in water samples under each detection mode. The amino acid column presents the top 5 amino acid metabolites with the highest release efficiency (water concentration/tissue concentration ratio), reflecting the capacity of these amino acids to transfer from skate meat to water.

	GC-MS	LC-MS		
Mode	POS	POS	NEG	Amino Acid
1	Lactic acid	Oleamide	Inosine	Taurine
2	Myo-Inositol	Trimethylamine N-oxide	Lactate	Glutamate
3	Arabitol	Linoleamide	Docosahexaenoic Acid	L-Isoleucine
4	Glucose	L-Phenylalanine	LysoPE	L-Phenylalanine
5	Glycerol	Hexylamine	Citric acid	L-Tyrosine

**Table 3:** Chemical attractant evaluation system.

Ranking	Test Substance	Behavioral Parameter Improvement Effects			Comprehensive Evaluation	
		Response Time Improvement Rate (%)	Distance Enhancement Rate (%)	Speed Enhancement Rate (%)	Comprehensive Score	Effect Level
1	Skate meat	70	515.4	746.2	94.8	Excellent
2	Mix	69	579.1	580.9	93.6	Excellent
3	Taurine	50	164.5	163	55.4	Moderate
4	Glu	44	33	33.3	38.5	Poor
5	Inositol	37	87.4	87.1	37.7	Poor
6	Lactate	3.3	20.6	20	21.7	Poor

## Data Availability

The original contributions presented in this study are included in the article/Supplementary Materials. Further inquiries can be directed to the corresponding authors.

## Supplementary Materials

The following supporting information can be downloaded at: https://www.mdpi.com/article/10.3390/biology14111627/s1, Code S1. Behavior Tracking Code.
