# Supplementary material for: Taurine-Dominated Feeding Attractant Mixture Induces Efficient Foraging in Neptunea cumingii
Source: Biology (Basel). 2025 Nov 19;14(11):1627. doi: 10.3390/biology14111627 (PMC12650022; doi:10.3390/biology14111627)
Supplement: Supplementary file 1 [file biology-14-01627-s001.zip › biology-3962467-supplementary.pdf]

Supplementary Code S1. Behavior Tracking Code

```
import cv2
import torch
import pandas as pd
from ultralytics import YOLO
from deep_sort_realtime.deepsort_tracker import DeepSort
from typing import List, Dict, Tuple, Any

class NeptuneaCumingiiTracker:
    """
    Neptunea cumingii movement detection and tracker.

    This implementation encapsulates YOLOv11 (or compatible models) for object detection,
    and DeepSORT algorithm for multi-object tracking. Its main function is to process
    video input and generate movement data containing timestamps, IDs, and spatial
    coordinates for each tracked Neptunea cumingii, ultimately outputting in CSV format.

    Attributes:
        video_path (str): Path to the input video file.
        model_path (str): Path to the YOLO model weights file (.pt).
        output_csv_path (str): Path to the output tracking data CSV file.
        yolo_model (YOLO): Loaded YOLO model instance.
        deepsort_tracker (DeepSort): Initialized DeepSORT tracker instance.
    """

    def __init__(self, video_path: str, model_path: str = 'best.pt', output_csv_path: str =
'reconstructed_raw_data.csv'):
        """
        Constructor for NeptuneaCumingiiTracker.

        Args:
            video_path (str): Path to the video file to be processed.
            model_path (str): Path to the trained YOLO model weights, defaults to 'best.pt'.
            output_csv_path (str): Path to save the tracking results CSV file.
        """
        self.video_path = video_path
        self.model_path = model_path
        self.output_csv_path = output_csv_path

        self.yolo_model = self._load_yolo_model()
        self.deepsort_tracker = self._initialize_tracker()

    def _load_yolo_model(self) -> YOLO:
        """Load YOLO model. If the specified path fails, attempt to load a pretrained model
```

```
as backup."""
        try:
            print(f"Loading YOLO model from '{self.model_path}'...")
            model = YOLO(self.model_path)
            print("Model loaded successfully.")
            return model
        except Exception as e:
            print(f"Failed to load model from '{self.model_path}': {e}")
            print("Attempting to load a pretrained 'yolov8n.pt' as a fallback...")
            try:
                model = YOLO('yolov8n.pt')
                print("Fallback model 'yolov8n.pt' loaded successfully.")
                return model
            except Exception as e_fallback:
                print(f"Failed to load fallback model: {e_fallback}")
                exit()

    def _initialize_tracker(self) -> DeepSort:
        """Initialize and return a configured DeepSORT tracker instance."""
        return DeepSort(
            max_age=30,                # Maximum number of unmatched frames before a
track is deleted
            n_init=3,                  # Number of consecutive detection frames required
to confirm a new track
            nms_max_overlap=1.0,       # Overlap threshold for non-maximum suppression
(NMS)
            max_cosine_distance=0.2,# Maximum cosine distance for appearance matching
            nn_budget=None,            # Memory budget for appearance descriptors
            embedder="mobilenet",      # Embedder model for feature extraction
            half=True,                 # Use half precision (FP16) for accelerated
computation
            bgr=True,                  # Whether input images are in BGR format
            embedder_gpu=True          # Run embedder on GPU
        )

    def run_tracking(self):
        """
        Execute the complete video processing pipeline, including frame-by-frame detection,
        tracking, and finally saving the results.
        """
        cap = cv2.VideoCapture(self.video_path)
        if not cap.isOpened():
            print(f"Error: Could not open video file {self.video_path}")
            return
```

```python
        tracking_results = []
        frame_count = 0

        while cap.isOpened():
            ret, frame = cap.read()
            if not ret:
                break

            # Get timestamp of the current frame
            timestamp = cap.get(cv2.CAP_PROP_POS_MSEC) / 1000.0

            # Process the current frame
            processed_tracks = self._process_frame(frame, timestamp)
            tracking_results.extend(processed_tracks)

            frame_count += 1
            if frame_count % 100 == 0:
                print(f"Processed {frame_count} frames...")

        cap.release()
        cv2.destroyAllWindows()

        self._save_results(tracking_results)

    def _process_frame(self, frame: Any, timestamp: float) -> List[Dict[str, Any]]:
        """Process a single frame, perform detection and tracking update, and return tracking
data for the current frame."""
        # 1. Perform object detection using YOLO model (assuming Neptunea cumingii class
ID is 0, confidence threshold 0.5)
        yolo_results = self.yolo_model.predict(frame, conf=0.5, classes=[0], verbose=False)

        detections = []
        for result in yolo_results:
            for box in result.boxes:
                x1, y1, x2, y2 = box.xyxy[0].tolist()
                confidence = box.conf[0].item()
                class_id = int(box.cls[0].item())
                # Convert to format required by DeepSORT: [[x_min, y_min, width,
height], confidence, class_id]
                detection_data = ([int(x1), int(y1), int(x2 - x1), int(y2 - y1)], confidence,
class_id)
                detections.append(detection_data)
```

```
        # 2. Update detection results to DeepSORT tracker
        tracks = self.deepsort_tracker.update_tracks(detections, frame=frame)

        # 3. Format output results for the current frame
        current_frame_tracks = []
        for track in tracks:
            if not track.is_confirmed() or track.time_since_update > 1:
                continue

            track_id = track.track_id
            ltrb = track.to_ltrb() # Get bounding box in left-top-right-bottom format
            center_x = (ltrb[0] + ltrb[2]) / 2
            center_y = (ltrb[1] + ltrb[3]) / 2

            current_frame_tracks.append({
                'timestamp': timestamp,
                'track_id': track_id,
                'center_x': center_x,
                'center_y': center_y,
                'confidence': track.get_det_conf(), # Get detection confidence associated
with this track
                'group_chinese': 'Glutamic Acid Group',    # Experimental group metadata
                'group_english': 'Glutamic_Acid',
            })
        return current_frame_tracks

    def _save_results(self, results: List[Dict[str, Any]]):
        """Aggregate all tracking results and save as a CSV file."""
        if not results:
            print("No tracking results were generated. Output file will not be created.")
            return

        df = pd.DataFrame(results)
        df.to_csv(self.output_csv_path, index=False)
        print(f"Tracking data successfully saved to: {self.output_csv_path}")

# --- Main program entry point ---
if __name__ == '__main__':
    # Define paths for video file and model weights
    VIDEO_FILE = 'experiment_video.mov' # Experimental video file to be processed
    MODEL_WEIGHTS = 'best.pt'                  # Trained YOLO model weights

    # Create tracker instance
    tracker_instance          =          NeptuneaCumingiiTracker(video_path=VIDEO_FILE,
```

```
model_path=MODEL_WEIGHTS)

    # Start tracking process
    tracker_instance.run_tracking()
```
